# Implant-Supported Overdentures: Current Status and Preclinical Testing of a Novel Attachment System

**DOI:** 10.3390/jcm12031012

**Published:** 2023-01-28

**Authors:** Frank Wendler, Lisa Diehl, Pejman Shayanfard, Matthias Karl

**Affiliations:** 1 Institute of Materials Simulation, Department of Materials Science, Friedrich-Alexander University Erlangen-Nürnberg, 90762 Fürth, Germany; 2 Department of Prosthodontics, Saarland University, 66421 Homburg, Germany

**Keywords:** implant-supported overdenture, patient satisfaction, retention, strain development, NiTi, wear, complications, superelasticity

## Abstract

Numerous attachment systems exist for implant-supported overdentures, with each having specific limitations in terms of retention, cost, wear, maintenance and cleanability. A retrospective analysis of patients restored with implant-supported overdentures using bars, telescopic crowns and Locator-type attachments was performed and the patients were interviewed. An in vitro strain gauge study compared telescopic crowns, Locator-type attachments and a novel flexible attachment system employing a shape memory alloy (NiTi) with respect to peri-implant strain development during insertion, loading and removal of an overdenture. A significantly lower number of attachment-related complications was observed in bars as compared to telescopic crowns (*p* = 0.00007) and Locator-type attachments (*p* = 0.00000), respectively. Greater overall patient satisfaction was noted in bar-retained restorations while Locator-type attachments led to lower levels of satisfaction regarding prosthesis retention. In vitro, telescopic crowns caused maximum strain development during prosthesis insertion and loading, while during removal this was observed in Locators with white retentive inserts. NiTi attachments caused significantly lower strain development during insertion as compared to telescopic crowns (*p* = 0.027). During loading, NiTi attachments caused significantly lower strain development than Locators with blue retentive inserts (*p* = 0.039). During removal, NiTi attachments caused significantly less strain development as compared to Locators with white retentive inserts (*p* = 0.027). Positional discrepancies between male and female attachment parts affected the retention and reaction force between both components, which may be minimized by using the novel NiTi attachment system. This may be beneficial in terms of component wear and implant loading.

## 1. Introduction

While not yet constituting the standard of care [1] in all countries, implant-retained removable prostheses have been shown to have a positive effect on functional and patient-centered outcomes [2,3,4]. In this context, a large body of literature comparing various implant and attachment configurations [5] does exist, at least partially reporting contradictory results.

There seems to be a consensus that despite the overall good performance, technical complications in such restorations are common [6], do occur more frequently as compared to fixed restorations [7,8], and correlate with the time in service [9]. Despite that, patients have been shown to have an unsatisfactory level of knowledge about potential complications [10].

Attachment-related problems, in particular the loss of retention due to wear at the retentive interface [9,11,12,13], as well as prosthesis fractures, predominantly occurring in the region of the attachment, seem to constitute the complications with the greatest incidence [14,15]. While some studies suggest that only minor differences in prosthodontic maintenance and peri-implant condition exist between different attachment systems [3,16], others claim that the type of attachment used would affect the treatment outcome [17,18]. As a trend, single standing attachments seem to be preferred over bar constructions due to their ease of cleaning [19,20], and prefabricated components comprising ball attachments and Locator-type attachments seem to be preferred over individually fabricated devices such as telescopic crowns [21] due to the lower initial cost [12,16].

Potential explanations for the rather rapid wear of the attachment systems may be seen in transfer inaccuracies between the clinical and the laboratory situation [22], as well as inter-implant angulation [23,24,25]. These findings from laboratory studies are supported by clinical results showing that the non-parallelism of the supporting implants resulted in a more frequent replacement of retention inserts [26].

Regardless of the specifics of any social security system, maintenance interventions cause costs that have to be seen in the context of the initial treatment costs, keeping in mind that removable restorations are often chosen when financial resources are limited. Several authors have already addressed this issue, showing that maxillary overdentures supported by four implants and retained by stud or bar attachments required 2.12 maintenance treatments per patient for the stud-retained group and 2.29 per patient for the bar-retained group over a period of three to nine years [20]. In patients with two mandibular implants, maintenance required on average 6.7 interventions per patient over a five-year period with the associated costs negatively influencing patient satisfaction [26]. A comparative study on mandibular implant overdentures showed that the initial costs constituted 75% of the total costs and were higher in the group with a bar on four implants, compared with the group with a bar on two implants and the group with two ball attachments, which required a significantly higher amount of aftercare, mostly for the re-adjustment of the retentive system [4]. Even a greater imbalance between initial and maintenance costs has been shown for mandibular overdentures with Locator-type attachments, for which the maintenance costs after five years were equal to or even higher than the initial cost of the treatment [12].

Apart from purely technical complications, biologic problems associated with implant therapy also have to be kept in mind. In this context, a recently published classification system for peri-implant defects also considers implant position as a relevant factor [27]. Despite the promising results described for regenerative treatment in peri-implant surgery, ref. [28] maintaining peri-implant health, e.g., by applying postbiotic substances, should be aimed for [29].

**Figure 1:**
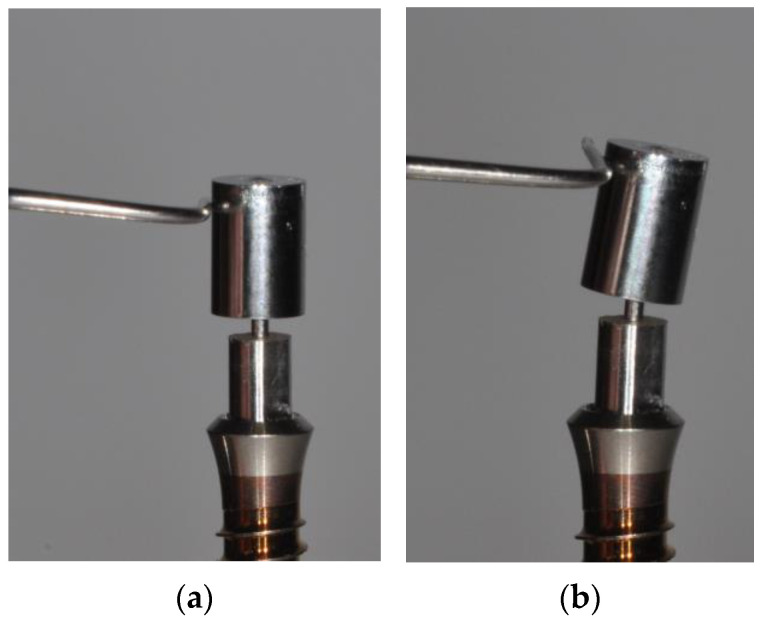
Prototype attachment system employing a cylindrical male attachment part connected to an abutment base via a 0.8 mm NiTi wire. (**a**) Attachment system mounted on an implant shoulder; (**b**) A horizontal force acting on the male attachment part causes a deflection relative to the base, which is supposed to compensate for misalignments of implants and transfer inaccuracies between patient situation and laboratory cast.

In order to overcome problems resulting from inter-implant angulation as well as from impression inaccuracies, a novel attachment system [30] incorporating a flexible element (Figure 1) made out of superelastic [31] Nickel–Titanium (NiTi) has been designed [32]. Despite concerns regarding the biocompatibility of Nickel-containing devices [33,34], NiTi components have been introduced in implant dentistry for retaining fixed or removable restorations [35,36,37,38,39] and have also been envisaged for eliminating gaps at the implant abutment interface [40].

The first goal of this study was to retrospectively evaluate complications occurring in edentulous patients treated with implant-retained removable prostheses based on bars, telescopic crowns and Locator-type attachments. The patients’ overall perception regarding the treatment rendered was studied by a questionnaire. In the second part, an in vitro biomechanical investigation was conducted, evaluating a prototype NiTi-based attachment system with respect to the passivity of fit, and the load transfer to the peri-implant bone using Locator and telescopic crowns for comparison.

## 2. Materials and Methods

### 2.1. Retrospective Analysis of Complications

**Figure 2:**
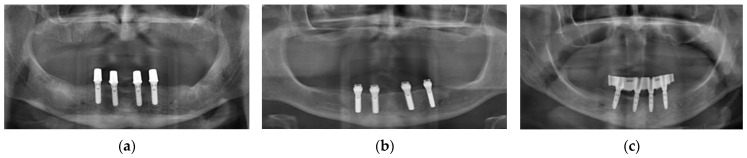
Typical patient situations with implant-supported overdentures analyzed in the first part of the study. (**a**) Telescopic crowns used as attachment system; (**b**) Locator-type attachments used as retentive elements; (**c**) Interforaminal implants splinted with a bar for superstructure retention.

Electronic patient charts were filtered for all patients who had received an implant-retained overdenture during the years 2006 to 2020 in the Department of Prosthodontics, Saarland University Dental School, Homburg, Germany. Patients with any teeth remaining in the jaw were excluded. All maintenance interventions directly related to the attachment system were recorded. Furthermore, only patients who had received bars, Locator-type attachments or telescopic crowns were included (Figure 2).

For comparative statistical analysis, the total numbers of complications recorded for the total numbers of implants used with a specific attachment system were considered. Neither the number of implants in a specific jaw nor the timepoint of the occurrence of a complication were considered. Assuming a binomial distribution of complications, Fisher’s exact test was applied for pairwise comparisons followed by the Holm–Bonferroni correction to compensate for multiple testing. All calculations were performed using the R software package (R, The R Foundation for Statistical Computing, Vienna, Austria; www.R-project.org; accessed on 16 December 2022) with the level of significance set at α = 0.05.

### 2.2. Patient Survey

All patients included in the retrospective chart analysis described above were requested to answer a series of questions as part of a regular recall appointment. Using a semi-structured questionnaire [10], patients were asked to rate their satisfaction with their prosthesis overall and the retention in particular on a scale from 1 (low) to 5 (high). In addition, they were asked to name the most significant problem experienced with their prosthesis, whether or not they had been informed about maintenance costs, and if they had been willing to pay a higher fee for initial treatment if complications could have been avoided.

Statistical analysis (R, The R Foundation for Statistical Computing, Vienna, Austria; www.R-project.org; accessed on 16 December 2022) of patients’ responses on satisfaction with overall treatment and with retention of the prostheses was based on pairwise comparisons using the Kruskal–Wallis rank sum test. Correction for multiple testing was performed using the Bonferroni method and the level of significance was set at α = 0.05 for all operations.

### 2.3. Testing of a Novel Attachment System

A clinical situation of an edentulous mandible with two interforaminal implants (Straumann Standard Plus 4.1 × 10.0 mm, Straumann AG, Basel Switzerland) was used for creating a realistic patient model out of denture resin (ProBase Cold, Ivoclar Vivadent, Schaan, Liechtenstein) including a gingival mask [25] with an approximate thickness of 2–3 mm (Adisil blau, Siladent, Goslar, Germany).

Five open-tray impressions were made using the respective screw-retained transfer copings, custom trays (PalatrayXL, Kulzer GmbH, Hanau, Germany) and polyether impression material (Impregum, 3MEspe, Seefeld, Germany). Definitive casts (Fujirock EP, GC Europe) incorporating implant analogs were then fabricated following standard laboratory procedures. On each master cast, three mandibular prostheses were fabricated using telescopic crowns, Locators (RN LOCATOR abutment tissue cuff height 1.0 mm, Straumann AG) and NiTi-based attachments (prototype, not commercially available), respectively.

The primary cylindrical telescopic crowns were fabricated on the basis of screw-retained burn-out plastic copings (RN synOcta Plastic Coping for synOcta 1.5; for crown, Straumann AG) using dental training alloy (Phantom-Metall NF, Dentsply Sirona Deutschland GmbH, Bensheim, Germany) employing standard casting and milling procedures. The secondary telescopic crowns were made from pattern resin (Pattern resin, GC Europe, Bad Homburg, Germany) and mounted in the prostheses using composite resin (Rebilda DC, Voco, Cuxhaven, Germany).

**Figure 3:**
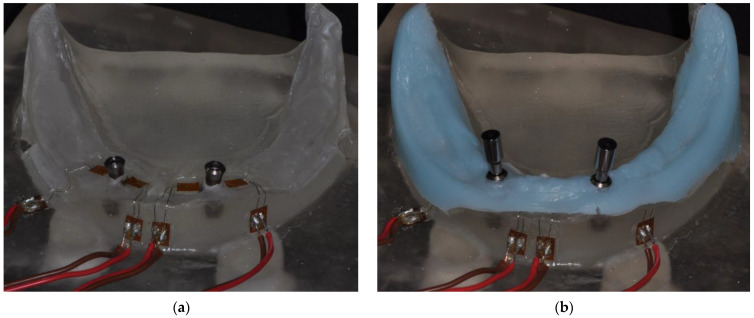
Existing patient situation with two interforaminal implants transferred to a resin model. (**a**) strain gauges were attached mesially and distally adjacent to the implants, capturing peri-implant strain development; (**b**) Model situation with gingival mask positioned and prototype abutments placed on the implants.

The male parts of the prototype NiTi-based attachments were assembled out of single components by a proprietary welding process allowing the maintenance of the superelasticity of the material (Admedes, Pforzheim, Germany). The NiTi rod connecting the base and retentive element had a diameter of 0.8 mm (Figure 3). Silicone rings were used as female parts and mounted in the prostheses using autopolymerizing resin (ProBase Cold, Ivoclar Vivadent, Schaan, Liechtenstein).

For the Locator prostheses, regular housings for retentive elements (Zest Dental Solutions, Carlsbad, CA, USA) were positioned in the prostheses following the manufacturer’s guidelines using autopolymerizing resin (ProBase Cold, Ivoclar Vivadent, Schaan, Liechtenstein). Female elements varying in retention force (blue 0.7 kg; pink 1.4 kg; white 2.3 kg) were positioned in the metal housings using the manufacturer’s service tool.

Linear strain gauges (LY11-0.6/120, 120Ω reference resistance, Hottinger Baldwin Messtechnik GmbH, Darmstadt, Germany) were attached to the model material mesially and distally adjacent to the implants [22] utilizing a measurement amplifier (Quantum X, Hottinger Baldwin Messtechnik GmbH, Darmstadt, Germany) and analyzing software (jBEAM, AMS GmbH, Chemnitz, Germany) for recording strains in the surroundings of the supporting implants at a sampling rate of 50/s.

**Figure 4:**
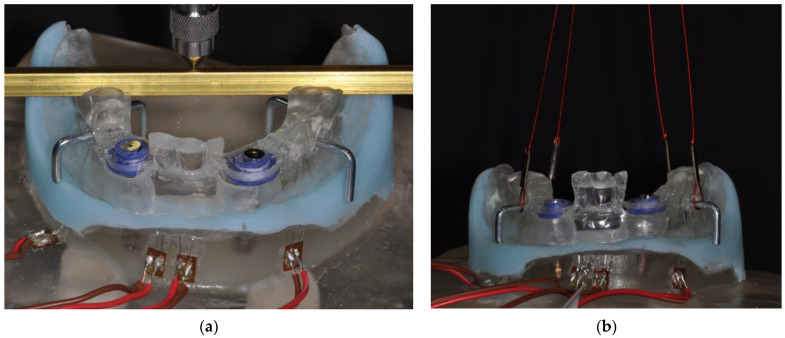
Measurement procedure. (**a**) Following placement of the prosthesis, masticatory static loading with 50 N was performed in the area of the second premolar/first molar; (**b**) As a final step, the prostheses were removed from the jaw model by applying axial force via wires.

**Figure 5:**
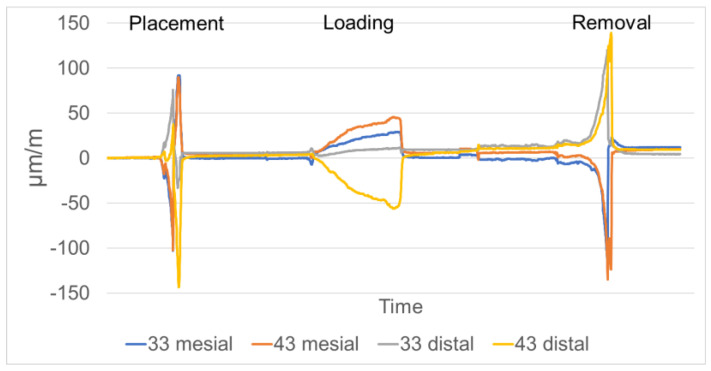
Strain development recorded at the peri-implant sensors (named according to their position relative to the implants: 33 mesial, 33 distal, 43 mesial, 43 distal) during seating, loading and removal of a specific prosthesis.

For determining the level of misfit strain generated by prosthesis insertion [22], the strain gauges were set to zero and the prostheses were manually positioned, ensuring the engagement of the attachments by applying finger pressure. Strain development in the peri-implant region as a consequence of masticatory loading of the prostheses was measured with the patient model positioned in a universal testing machine (Z020, Zwick/Roell, Ulm, Germany), applying a static load of 50 N (Figure 4a) in the second premolar/first molar region. As a final step, the prostheses were removed from the supporting implants in the universal testing machine (Figure 4b), recording maximum separation force and strain development in the peri-implant area (Figure 5).

Comparative statistical analysis (R, The R Foundation for Statistical Computing, Vienna, Austria; www.R-project.org; accessed on 16 December 2022) was based on absolute mean strain values recorded at the four sensors mesially and distally adjacent to the implants. In subsequent order, the statistical tests applied were Shapiro–Wilk normality test, Levene’s test for homogeneity of variance, Kruskal–Wallis test for nonparametric one-way analysis, and pairwise comparisons using Nemenyi’s all-pairs test. Bonferroni correction was carried out in order to compensate for multiple testing and the level of significance was set at α = 0.05 for all operations.

## 3. Results

### 3.1. Retrospective Analysis of Complications

During the period from 2006 to 2020, a total of 78 edentulous jaws had been restored with implant supported overdentures and were included for analysis (Table 1). The mean number of implants used per jaw ranged from 3.4 for Locator-type attachments to 4.2 for bar constructions and 4.5 for telescopic crowns. Only one implant was lost in the group of bar-retained overdentures, while three implants were lost in Locator-type prostheses, and a total of five implants were lost in the group of telescopic crowns.

Only nine attachment-related complications were observed in bar-retained restorations, which was significantly lower as compared to telescopic crowns, where 34 complications occurred (*p* = 0.00007). The greatest number of complications occurred with Locator-type attachments, requiring 56 interventions, which was significantly more as compared to telescopic crowns (*p* = 0.0004) and bars (*p* = 0.00000), respectively.

The most frequent complication in telescopic crowns was the decementation of primary crowns, followed by an adjustment of friction, and the greatest number of complications recorded in one jaw was sixteen. Apart from that, in one patient with telescopic crowns, an extremely high frequency of denture tooth fractures occurred. The replacement of retentive clips was the most frequent intervention in bar-retained overdentures, but no cumulation of complications occurred in these patients. The replacement of plastic inserts and loosening of male attachment parts were the most frequent complications observed in Locator-type attachments, and the greatest number of complications recorded in one jaw was ten. Despite using single standing Locator-type attachments, one patient was not able to adequately clean the supporting implants, requiring repetitive professional cleanings.

In summary, despite comparable patient characteristics, the use of Locators as attachment systems led to the greatest number of attachment-related complications as compared to individually fabricated bars or telescopic crowns.

### 3.2. Patient Survey

Only 2 out of 19 patients having received telescopic-crown-retained overdentures participated in the survey, while 7 out of 25 patients with Locator-type attachments and 5 out of 21 patients with bar-retained prostheses could be interviewed.

Out of the 14 survey participants only 4 had been informed upfront about the maintenance cost of their removable restorations, and 8 patients would have been willing to pay higher initial treatment costs if complications and maintenance needs could have been avoided or if the retention of the prosthesis could have already been optimized upon delivery. In the groups of patients restored with bars and telescopic crowns, only one patient complained about a somewhat bulky restoration, while the others claimed not to have any major problem with the restoration. Out of the seven patients restored with Locator-type attachments, five described a lack of retention or repeated changes in the female attachment parts as the main problem.

**Figure 6:**
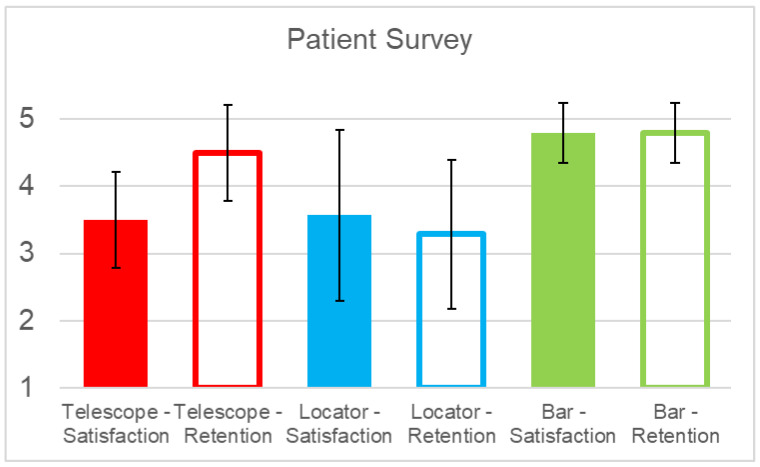
Patient satisfaction with respect to overall treatment and retention of implant-supported overdentures employing telescopic crowns, Locator-type attachments, and bars as retentive devices. Satisfaction could be rated on a scale from 1 (low) to 5 (high).

On a scale of 1–5, the mean overall satisfaction ranged from 3.50 for telescopic crowns to 3.57 for Locator-type attachments and 4.80 for bars (Figure 6). While no statistically significant difference could be observed between the attachment systems employed (Table 2), a trend towards greater satisfaction with bar-retained restorations was noted (overall satisfaction: bar vs. telescope *p* = 0.052; bar vs. Locator *p* = 0.070). Similarly, no significant difference in satisfaction with regards to retention was observed between the different attachment systems. However, Locator-type attachments showed a trend towards lower levels of satisfaction, which in comparison with bars (*p* = 0.022) was statistically significant prior to the Bonferroni correction for multiple testing (Table 2).

Overall, the greatest consistency in the treatment outcome was seen in bar-retained restorations, which is evidenced by comparably low standard deviations for overall satisfaction and prosthesis retention. The greatest variation was observed in prostheses employing Locator attachments.

### 3.3. Testing of a Novel Attachment System

**Figure 7:**
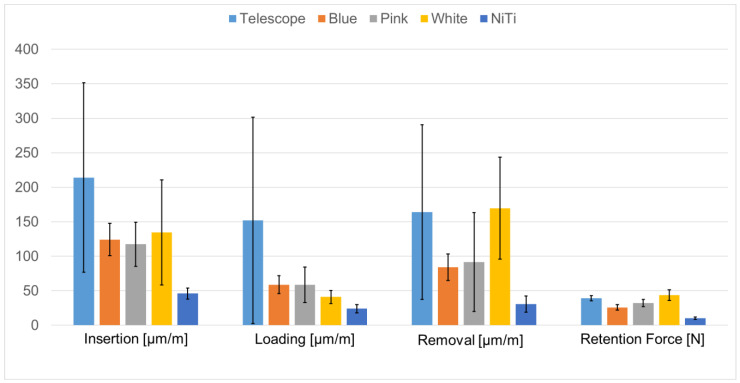
Mean of absolute strain values calculated for each attachment system and for each loading scenario (insertion, loading, removal) in addition to the mean retention forces of the prostheses.

The means of the absolute strain values were calculated for each attachment system and for each loading scenario (insertion, loading, removal), which are shown in Figure 7, in addition to the mean retention forces. Prostheses retained by telescopic crowns showed maximum strain development in all loading scenarios with the exception of prosthesis removal, where prostheses with white Locator inserts exhibited a slightly larger mean value. Additionally, the standard deviations calculated in telescopic-crown-retained prostheses were much greater as compared to all other groups. The NiTi attachments showed the lowest mean values for all the parameters recorded.

The Shapiro–Wilk normality test showed significant values for the loading scenarios of insertion (*p* = 0.012) and loading (*p* = 0.000), while no significant values were seen for removal (*p* = 0.354) or retention force (*p* = 0.629). Similarly, Levene’s test for homogeneity of variances indicated a non-normal distribution of values and a non-homogeneity of variances for the loading scenarios of insertion (*p* = 0.016) and loading (*p* = 0.072), while no significant values were seen for removal (*p* = 0.066) or retention force (*p* = 0.472). The non-parametric one-way analysis applying the Kruskal–Wallis test showed significant values for all the parameters (insertion *p* = 0.012; loading *p* = 0.009; removal *p* = 0.012; retention force *p* = 0.000). The results of the subsequent pairwise comparisons are given in Table 3.

Telescopic crowns caused maximum strain development during insertion, reaching a mean value of 214.17µm/m, which was significantly greater as compared to the NiTi attachments (45.94 µm/m; *p* = 0.027). During loading, maximum mean strains of 152.16 µm/m were again seen in telescopic crowns, which were significantly greater as compared to Locators with blue retentive inserts (58.86 µm/m; *p* = 0.009). While the difference between telescopic crowns and the NiTi attachments during loading was not significant (*p* = 0.074), the comparison between the NiTi attachments and Locators with blue retentive inserts reached a significant value (*p* = 0.039). During removal, the prostheses with NiTi attachments caused significantly less strain development as compared to those with Locators with white retentive inserts (*p* = 0.027). An increase in retention force for Locator attachments from blue to pink to white as indicated by the manufacturer was observed, although the differences were not significant; however, a noticeable increase in strain development during prosthesis removal was seen when white retentive inserts were used. The NiTi attachments showed significantly lower retention values as compared to telescopic crowns (*p* = 0.021) and Locators with white retentive inserts (*p* = 0.004).

The novel attachment system under all the loading scenarios led to the lowest strain recordings in the peri-implant area while maintaining prosthesis retention. During placement and removal, the flexibility of the male attachment part allowed for a common path of draw, while during loading, the settling of the prosthesis was accomplished by the flexing of the attachment system.

## 4. Discussion

Given the variety of treatment concepts, personal preferences, and socioeconomic settings, numerous authors have examined the clinical performance of attachment systems for implant-supported overdentures [3,5,16,17,18]. As such, the retrospective clinical data presented here is rather confirmatory and seems to be in line with the current knowledge despite the inhomogeneity of the patients analyzed in terms of implant brand, distribution of implants, attachment system, and opposing dentition. With the focus of this paper being on the attachment system, only specific complications were reported, but of course unrelated problems such as fractures of denture teeth also occurred in the small cohort studied here. Furthermore, we did not report on biologic problems associated with implant therapy leading to destruction of both hard and soft tissues as well as a maintenance regimen [27,28,29].

The use of bars as the attachment system led to the lowest number of technical complications [20], and hence to the highest level of patient satisfaction with both the overall treatment and retention of the prostheses. Despite constituting a labor- and cost-intensive option [12,16,21] similar to bars, telescopic crowns led to a much greater number of complications, which was surpassed by prefabricated Locator-type attachments, with the loss of retention by far constituting the major problem [9,11,12,13]. This also led to a significantly lower level of patient satisfaction [26].

From an industry perspective, replacing female retentive inserts might be seen as being positive and generating revenue. From a clinician’s perspective, maintenance interventions are rarely profitable but often cause organizational problems with respect to patient scheduling, and the patients often are frustrated. Although a meaningful cost analysis was not possible based on the comparably small number of patients treated over a period of 15 years using a variety of dental laboratories and materials, it became obvious that patients had an unsatisfactory level of knowledge about potential complications and maintenance needs, including the associated costs [10,13]. Two clinical studies [4,12] have shown that maintenance costs reach considerable amounts relative to the initial treatment costs.

It may be seen as a further limitation of this retrospective clinical study that patients who had received implants supplementing natural abutments for the retention of removable restorations were not considered. Additionally, the questionnaire given to the patients was limited to technical aspects of the prostheses instead of using, e.g., the OHIP questionnaire [41].

Derived from conventional, tooth-supported, removable restorations, telescopic crowns are quite popular in some countries as attachment systems for implant-supported overdentures. Setting the retention of telescopic crowns is demanding, requires experience, and seems to differ drastically between tooth-supported and implant-supported restorations. On natural teeth, a drastic decrease in retention can be seen a few days after delivery following tooth movement directed by the removable prosthesis. Due to the lack of a periodontal ligament, such extensive movement seems not to occur when dental implants are being used as abutments for removable restorations [42]. Consequently, reducing the friction of telescopic crown attachments constituted a frequent problem in the patient cohort considered, which is in accordance with previous reports [17,19].

From a biomechanical perspective, the ability of attachment systems in transferring misfit loads and masticatory loads to the supporting implants and bone also has to be considered. Rigid attachment systems have been shown in independent in vitro studies to transfer potentially critical amounts of moment loads to the supporting implants during the masticatory loading of the removable prostheses [43,44]. Based on a clinical study using two implants to restore the edentulous maxilla and showing compromised outcomes, it may be inferred that mechanical overloading due to stiffness of the telescopic crowns used was a critical co-factor [45]. Similarly, a clinical study using four maxillary implants and Locator-type attachments reported an implant survival rate of only 86.2% after a one-year observation period, while the mean peri-implant bone loss was 1.01 ± 0.77 [41].

In an attempt to explain the clinical performance of telescopic crowns and Locator-type attachments as well as to search for solutions to the problems described above, the in vitro strain gauge study tried to compare these attachment systems. As hypothesized above, the stiffness of the attachment system obviously had an effect on the retention force and peri-implant strain development, as telescopic crowns and Locator attachments with white retentive inserts (greatest retention examined here) showed maximum values. As expected, the individually fabricated telescopic crowns showed the largest standard deviations, indicating that adjusting the retention is demanding in these attachments.

A trend of increased retention force was seen in the Locator-type attachments from blue to pink to white retention inserts, reflecting the manufacturer’s information. Interestingly, this increase in retention force did cause an increase in peri-implant strain during prosthesis removal, but no clear trend was seen during prosthesis insertion and loading. It was noted that the retention force measured in Locator prostheses with blue and pink retention inserts was higher than the retention force expected based on the manufacturer’s data (Table 4).

It may be argued that positional mismatch between female and male attachment parts resulting from inevitable fabrication errors as well as the non-parallelism of the supporting implants may have caused this discrepancy. When using inserts with maximum retention, this effect may be hidden. Based on experience, patients remove their prostheses two to three times per day on average, making this a relevant loading scenario that may be detrimental not only to the attachment system but also to the implant. While wear at the retentive interface of the attachment system [24,25] was not tested as part of this study, it may be argued that changes in separating force may be caused by wear resulting from positional mismatch between male and female parts.

The novel NiTi-based attachment system showed retention values not significantly differing from Locator-type attachments with blue or pink retention inserts, but drastically reduced strain development during all loading scenarios, showing the lowest mean values for all parameters recorded. As it was not possible to experimentally determine the reaction force between the male and female parts of the attachment systems used here, the strains recorded in the surroundings of the supporting implants were used as a surrogate measure. Given the lower strain values in the NiTi-based attachments, it may be expected that wear phenomena of the attachment system can be reduced by providing sufficient lateral flexibility of the male part. Of course, additional and more sophisticated investigations addressing the long-term stability of such an attachment system, as well as its biocompatibility [33,34], are needed. Comparable to the situation of NiTi-based endodontic instruments, repeated deformation of the attachment may fatigue the material and ultimately lead to fractures. As it was the purpose of this study to test the basic applicability, fatigue testing has not yet been performed.

Besides the in vitro nature of the investigation based on one specific patient situation, several limitations have to be taken into account. The NiTi attachment represented the first prototype, requiring far too much space for clinical use, as the exact dimensions of the flexible element could not yet be properly set due to a lack of experimental data. While best representing clinical reality, finger pressure during prosthesis insertion could not be standardized and may have affected the strain readings. Similarly, despite a common vertical path of insertion having been established by adjusting the model base, it may be argued that the peak loading of implants was related to an offset direction of pull during prosthesis removal. Similar to variations in patient anatomy and implant positions, numerous loading positions do occur under clinical conditions. Based on a previous experiment [30], loading in the molar/premolar area seemed to be most relevant and hence was chosen as the sole loading scenario. Assuming that compressive and tensile strain are equally detrimental to the attachment system, implant components and alveolar bone, the means of the absolute strain values were calculated per attachment system. This approach not only led to high standard deviations affecting comparative statistics but also only allowed for comparisons on a relative scale.

## 5. Conclusions

Based on the findings presented, it can be concluded for implant-supported overdentures that:

1. Locator-type attachments are problematic with respect to adjusting and maintaining retention;
2. Individually fabricated telescopic crowns do not warrant the higher laboratory costs associated with them and are problematic in terms of adjusting retention as well as in transferring loads to the supporting implants;
3. While yet at an early, preclinical stage, the flexible NiTi attachment tested may constitute an alternative solution with well-controlled retention and reduced wear at the male/female attachment interface;
4. Apart from technical aspects, biologic complications associated with implant therapy continue to be problematic and should be monitored during the maintenance phase.

## Figures and Tables

**Table 1:** Results of retrospective patient analysis.

		Telescope	Locator	Bar
Number of jaws		24	29	25
Number of implants		109	100	104
Mean number of implants per jaw		4.5 (±0.9)	3.4 (±1.3)	4.2 (±0.5)
Implants lost during observation period	T < 90 d	1	1	0
	90 d < T < 365 d	0	0	0
	365 d < T < 730 d	0	1	0
	T > 730 d	4	1	1
Attachment associated complications	Total number	34	56	9
	T < 90 d	9	16	2
	90 d < T < 365 d	4	7	2
	365 d < T < 730 d	5	8	0
	T > 730 d	16	25	5
	most frequent	Decementation of primary telescopic crown	Replacement of insert	Replacement of clips
	second most frequent	Adjustment of friction⑩(Reduction)	Loosening/Loss of Locator abutment	Activation of clips/Screw loosening
Remark		16 complications occurred in one specific prosthesis	10 complications occurred in one specific prosthesis	No cumulation of complications in a specific prosthesis

**Table 2:** Results (*p*-value) of pairwise comparisons (Kruskal–Wallis rank sum test; Bonferroni correction) between patient satisfaction with respect to overall treatment and retention of implant-supported overdentures employing telescopic crowns, Locator-type attachments, and bars as retentive devices.

	Telescope		Locator		Bar	
	Overall Satisfaction					
	*p*-value	corrected *p*-value	*p*-value	corrected *p*-value	*p*-value	corrected *p*-value
Telescope	-	-	0.880	0.880	0.052	0.156
Locator	0.175	0.349	-	-	0.070	0.156
Bar	0.462	0.462	0.022 *	0.065	-	-
	**Retention**					

Level of significance set at α = 0.05; significant differences are marked with *.

**Table 3:** Results (*p*-value) of pairwise comparisons (Nemenyi’s all-pairs test; Bonferroni correction) between the attachment systems tested for prosthesis placement (a), loading (b), prosthesis removal (c) and retention force (d).

**(a) Prosthesis Placement**					
	**Telescope**	**Locator Blue**	**Locator Pink**	**Locator White**	**NiTi**
Telescope	-	0.989	0.937	0.900	0.027 *
Locator Blue		-	0.998	0.994	0.109
Locator Pink			-	1.000	0.215
Locator White				-	0.269
NiTi					-
**(b) Loading**					
	**Telescope**	**Locator Blue**	**Locator Pink**	**Locator White**	**NiTi**
Telescope	-	0.009 *	0.999	0.777	0.074
Locator Blue		-	0.989	0.640	0.039 *
Locator Pink			-	0.900	0.143
Locator White				-	0.640
NiTi					-
**(c) Prosthesis Removal**					
	**Telescope**	**Locator Blue**	**Locator Pink**	**Locator White**	**NiTi**
Telescope	-	0.925	0.854	0.989	0.109
Locator Blue		-	1.000	0.688	0.516
Locator Pink			-	0.566	0.640
Locator White				-	0.027 *
NiTi					-
**(d) Retention force**					
	**Telescope**	**Locator Blue**	**Locator Pink**	**Locator White**	**NiTi**
Telescope	-	0.318	0.836	0.991	0.021 *
Locator Blue		-	0.919	0.119	0.827
Locator Pink			-	0.553	0.308
Locator White				-	0.004 *
NiTi					-

Level of significance set at α = 0.05; significant differences are marked with *.

**Table 4:** Comparison of retention forces expected based on manufacturer’s data and values recorded experimentally.

	Locator Blue	Locator Pink	Locator White
Manufacturer information	0.7 kg	1.4 kg	2.3.kg
Expected for 2 attachments	13.73 N	27.46 N	45.12 N
Recorded (Figure 7)	25.92 N	32.12 N	43.72 N

## Data Availability

The data presented in this study are available on request from the corresponding author.
